# Detection of Rat Hepatitis E Virus in Pigs, Spain, 2023

**DOI:** 10.3201/eid3004.231629

**Published:** 2024-04

**Authors:** Lucia Rios-Muñoz, Moisés Gonzálvez, Javier Caballero-Gomez, Sabrina Castro-Scholten, María Casares-Jimenez, Irene Agulló-Ros, Diana Corona-Mata, Ignacio García-Bocanegra, Pedro Lopez-Lopez, Tomás Fajardo, João R. Mesquita, María A. Risalde, Antonio Rivero-Juarez, Antonio Rivero

**Affiliations:** Universidad de Córdoba, Córdoba, Spain (L. Rios-Muñoz, M. Gonzálvez, J. Caballero-Gomez, S. Castro-Scholten, I. Agulló-Ros, I. García-Bocanegra, T. Fajardo, M.A. Risalde);; Instituto Maimónides de Investigación Biomédica de Córdoba (L. Rios-Muñoz, J. Caballero-Gomez, M. Casares-Jimenez, D. Corona-Mata, P. Lopez-Lopez, A. Rivero-Juarez, A. Rivero);; Hospital Universitario Reina Sofía, Córdoba, Spain (L. Rios-Muñoz, J. Caballero-Gomez, M. Casares-Jimenez, D. Corona-Mata, P. Lopez-Lopez, A. Rivero-Juarez);; Universidad de Murcia, Murcia, Spain (M. Gonzálvez);; Instituto de Salud Carlos III, Madrid, Spain (J. Caballero-Gomez, M. Casares-Jimenez, D. Corona-Mata, I. García-Bocanegra, P. Lopez-Lopez, M.A. Risalde, A. Rivero-Juarez, A. Rivero);; Universidade do Porto and Laboratório para a Investigação Integrativa e Translacional em Saúde Populacional, Porto, Portugal (J.R. Mesquita)

**Keywords:** Rat hepatitis E virus, viruses, zoonoses, food safety, pigs, Spain

## Abstract

We identified rat hepatitis E virus (HEV) RNA in farmed pigs from Spain. Our results indicate that pigs might be susceptible to rat HEV and could serve as viral intermediaries between rodents and humans. Europe should evaluate the prevalence of rat HEV in farmed pigs to assess the risk to public health.

Hepatitis E virus (HEV) is a major cause of acute viral hepatitis in Europe. HEV is classified into 8 major genotypes, but zoonotic genotype 3 is the most prevalent on the continent (*1*). HEV was considered the only zoonotic species in the Hepeviridae family until rat HEV (*Rocahepevirus ratti*) was identified. Rat HEV was the causal agent of chronic hepatitis in a transplant recipient from Hong Kong in 2018 (*2*). Since that discovery, nearly 30 cases of chronic and acute hepatitis have been reported in America, Asia, and Europe (*3*–*6*), affecting both immunosuppressed and immunocompetent persons. Those cases highlight the zoonotic potential of rat HEV, emphasizing its growing concern to public health.

Rodents are the main host of rat HEV, but its transmission routes remain unclear. Although direct and indirect contact with rodents have been suggested as potential transmission routes, only 1 registered case has involved such contact (*6*). Thus, alternative sources of infection seem possible, potentially from an alternate host with which humans have more contact (*7*). Because domestic pigs (*Sus scrofa domestica*) are highly susceptible to HEV and constitute the main natural viral reservoir, they could also be susceptible to rat HEV and potentially serve as hosts. Confirming that hypothesis could have major implications for public health. We aimed to assess the presence of rat HEV in a population of farmed pigs in Spain.

During May–June 2023, we randomly selected and prospectively sampled domestic pigs from 5 intensive breeding system farms in Cordoba, southern Spain. We collected rectal fecal samples from each pig and stored samples at −80°C until RNA extraction (Appendix).

We included a total of 387 pigs in the study and found rat HEV in 44 pigs, an individual prevalence of 11.4% (95% CI 8.6%–14.9%) (Table). Sequencing confirmed the identity as rat HEV (species *R. ratti)* (GenBank accession nos. OR977681­–7 and OR977689­–7711) (Appendix Figures 1, 2). Among the 5 farms, 2 (40%) had >1 rat HEV–positive pig. Of note, 93.2% (41/44) of positive animals were from the same farm (Figure; Appendix Table 3). HEV RNA was detected in 6 pigs, indicating a prevalence of 1.6% (95% CI 0.6%–3.4%). All HEV-positive pigs were from the same farm and had sequences consistent with HEV genotype 3f (GenBank accession nos. OR818554–60), but rat HEV was not detected in that farm.

**Table:** Demographic data of pigs and characteristics of farms in a study of detection of rat HEV in pigs, Spain, 2023*

Characteristics	No. (%), n = 387
Age range	
Adult	188 (48.6)
Subadult	169 (43.7)
Unknown	30 (7.8)
Breed	
Iberian	159 (41.1)
White	148 (38.2)
Iberian cross	80 (20.7)
Aptitude	
Reproductive	188 (48.6)
Fattening	199 (51.4)
Farm HEV status	
Rat HEV–positive	44 (11.4)
HEV-positive	6 (1.6)

*HEV, hepatitis E virus.

**Figure:**
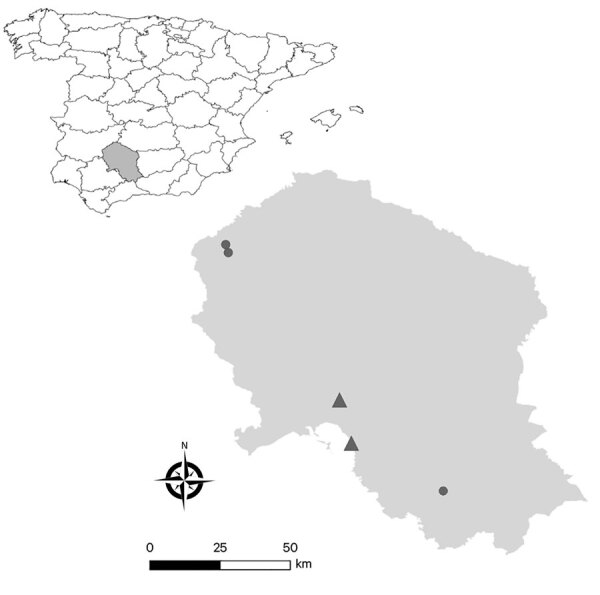
Geographic locations of farms included in a study of rat hepatitis E virus in pigs, Spain, 2023. Triangles indicate farms with >1 pig positive for rat HEV RNA are marked, circles farms with no positive pigs. Inset shows shaded area in Spain where the sampling occurred.

The hypothesis that pigs are not susceptible to rat HEV was formed on the basis of experimental in vivo studies (*8*). Because animals in that study were not infected after challenge with rat HEV strains (*8*), it appeared that pigs were resistant to rat HEV infection. However, our study detected rat HEV RNA in pigs, suggesting the possible role of pigs in rat HEV epidemiology. That finding increases the range of species susceptible to rat HEV, suggesting that its transmission might not be restricted to rodents. The number of positive animals we found suggests that rat HEV is widespread among pig populations in the study area. That observation might be linked to the elevated positivity rate (55%) discovered in rodents from the same region (*9*), implying that the lack of rodent control measures might increase the risk for rat HEV transmission.

The presence of rat HEV in farmed pigs is of public health concern, especially considering global pork consumption. Our study highlights the possibility that pigs intended for human consumption could contribute to rat HEV transmission. The European Food Safety Authority (EFSA) recommends monitoring HEV in pigs to identify alterations in virus distribution and prevent its spread to new farms, aiming to reduce human cases (*10*). Our results suggest that a preliminary evaluation of rat HEV in farmed pigs should be also conducted in Europe, which could confirm our results and increase our understanding of virus transmission.

The first limitation of our study is that because of its exploratory nature, the sampling area was restricted to a single region, but our findings underscore the need to extend the evaluation of rat HEV to determine its magnitude. Second, because no serologic assays are available for detecting rat HEV antibodies in pigs, our analysis was limited to molecular testing on fecal samples; thus, we cannot confirm rat HEV infection. However, our study justifies the design of new studies to evaluate the presence of rat HEV in blood and tissues samples. Finally, implementation of serologic analysis on rat HEV might enhance our comprehension of the pathogenesis of both HEV and rat HEV and assist in future investigations into risk factors.

In conclusion, our study shows the possibility that pigs are susceptible to rat HEV infection, challenging previous assumptions. Further studies are warranted to determine the role of pigs in rat HEV epidemiology and to assess the risk for direct or indirect zoonotic transmission from pigs. In addition, Europe should conduct an evaluation of rat HEV in farmed pigs to assess the overall risk to public health.

Appendix Additional information on detection of rat hepatitis E virus in pigs, Spain, 2023.
