# Supplementary material for: Detection of Rat Hepatitis E Virus in Pigs, Spain, 2023
Source: Emerg Infect Dis. 2024 Apr;30(4):823–6. doi: 10.3201/eid3004.231629 (PMC10977822; doi:10.3201/eid3004.231629)
Supplement: Appendix — Additional information on detection of rat hepatitis E virus in pigs, Spain, 2023. [file 23-1629-Techapp-s1.pdf]

Article DOI: https://doi.org/10.3201/eid3004.231629

EID cannot ensure accessibility for supplementary materials supplied by authors. Readers who have difficulty accessing supplementary content should contact the authors for assistance.

Detection of Rat Hepatitis E Virus in Pigs, Spain, 2023

Appendix

Animal and Farm Sampling

Intensive pig farms included in the present study were selected by simple random sampling from official flock registers obtained from the Regional Government of Andalusia. The sample size was calculated assuming a herd prevalence of 50%, which provides the highest sample size in studies based on unknown prevalence, with a 95% CI and accepted error of 5%, giving 385 animals to be sampled (*1*). Within each farm, a mean of 75 (range 71–80) animals were sampled by systematic random sampling, with the objective of detecting rat hepatitis E virus (rat HEV) infection with a probability of 95% and a minimum expected prevalence of 4% (*2*).

Rat HEV Molecular Evaluation

Viral RNA extraction from 0.25 mg of feces was performed by diluting feces in 300 µL of PBS and processed using the IndiSpin Pathogen Kit (formerly known as QIAamp Cador Pathogen Mini Kit) using the QIAcube (QIAGEN, Hilden, Germany) automatic procedure. RNA was then eluted in 50 µL.

All individuals underwent real-time quantitative PCR (qPCR) testing for HEV and rat HEV. For HEV evaluation, a qPCR assay previously developed and validated by our group was employed (*3*), using the 1st WHO standard for acid nucleic amplification–based HEV RNA assays (supplied by the Paul-Ehrlich-Institut under the code PEI 6219/10) as a positive control. To detect rat HEV RNA, we used a PCR targeting the 5′ untranslated region (5′UTR) (*4*). A rat liver sample from a rodent identified in our lab (GenBank accession no. OR282813) was used as

positive control. Samples positive for HEV, rat HEV, or both, underwent sequencing. In cases of HEV-positive samples, a nested PCR targeting a 420 nt segment of ORF2 was conducted (*3*). The approach for sequencing rat HEV–positive samples involved three nested PCRs, targeting three regions located on the ORF1. The regions had lengths of 880 bp (*5*), 220 bp, and 230 bp, respectively.

References

1. Thrusfield M. Veterinary Epidemiology, fourth ed. Oxford, UK: Wiley Backweel; 2018.

2. Ryll R, Bernstein S, Heuser E, Schlegel M, Dremsek P, Zumpe M, et al. Detection of rat hepatitis E virus in wild Norway rats (*Rattus norvegicus*) and black rats (*Rattus rattus*) from 11 European countries. Vet Microbiol. 2017;208:58–68. PubMed https://doi.org/10.1016/j.vetmic.2017.07.001

3. Frías M, López-López P, Zafra I, Caballero-Gómez J, Machuca I, Camacho Á, et al. Development and clinical validation of a pangenotypic PCR-based assay for the detection and quantification of hepatitis E virus (*Orthohepevirus* A genus). J Clin Microbiol. 2021;59:e02075-20. PubMed https://doi.org/10.1128/JCM.02075-20

4. Parraud D, Lhomme S, Péron JM, Da Silva I, Tavitian S, Kamar N, et al. Rat hepatitis E virus: presence in humans in south-western France? Front Med (Lausanne). 2021;8:726363. PubMed https://doi.org/10.3389/fmed.2021.726363

5. Mulyanto, Depamede SN, Sriasih M, Takahashi M, Nagashima S, Jirintai S, et al. Frequent detection and characterization of hepatitis E virus variants in wild rats (*Rattus rattus*) in Indonesia. Arch Virol. 2013;158:87–96. PubMed https://doi.org/10.1007/s00705-012-1462-0

Appendix Table 1. Primer and probe sets used for the detection of rat hepatitis E virus RNA*

HEV	PCR type	Forward primer, 5′→3′	Reverse primer, 5′→3′	Probe, 5′→3′	Ref.
HEV_qPCR	qPCR	RGTRGTTTCTGGGGTGAC	AKGGRTTGGTTGGRTGA	5′-FAM-TGAYTCYCARCCCTTCGC-TAMRA-3′	(*3*)
HEV_ORF2	Nested	CAAGGHTGGCGYTCKGTTGAGAC	CCCTTRTCCTGCTGAGCRTTCTC		(*3*)
		GYTCKGTTGAGACCWCBGGBGT	TTMACCWGTCRGCTCGCCATTGGC		
RatHEV					
RatHEV_Parraud	qPCR	CCACGGGGTTAATACTGC	CGGATGCGACCAAGAAACAG	5′-6FAM-CGGCTACCGCCTTTGCTAATGC-BBQ-3′	(*4*)
RatHEV_Mulyanto	Nested	CCTYTGCAGCTTGTCTTTGA	ATGCGTGCTCATGGHATG		(*5*)
		CTGTTTCTTGGTCGCATCC	CTGATCTTTCCTTTTGCAC		
RatHEV_B	Nested	TTTGCTAATGCTCAGGTGGT	ATGCGTGCTCATGGHATG		This study
		CTGTTTCTTGGTCGCATCCG	AACATCCGCCGTTGCATTCTT		
RatHEV_E	Nested	CCTYTGCAGCTTGTCTTTGA	ATGCGTGCTCATGGHATG		This study
		CTGTTTCTTGGTCGCATCC	CTGATCTTTCCTTTTGCAC		

*Underlined letters indicate degenerate primers. qPCR, quantitative PCR; Ref., reference.

Appendix Table 2. Thermocycle conditions

Step	No. cycles	Time, s	Temperature					
			HEV qPCR	HEV ORF2	RatHEV Parraud	RatHEV Mulyanto	RatHEV B	RatHEV E
1st PCR								
UNG Activity		600	25°C	25°C	25°C	25°C	25°C	25°C
Retrotranscription		300	52°C	52°C	52°C	52°C	52°C	52°C
Denaturalization		10	95°C	95°C	95°C	95°C	95°C	95°C
Denaturalization	x45	5	95°C	95°C	95°C	95°C	95°C	95°C
Annealing		30	58°C	51°C	58°C	58°C	58°C	58°C
2nd PCR								
Denaturalization		120		95°C		95°C	95°C	95°C
Denaturalization	x45	60		95°C		95°C	95°C	95°C
Annealing		60		52°C		58°C	58°C	58°C
Extension		60		72°C		72°C	72°C	72°C
Final extension		300		72°C		72°C	72°C	72°C

*HEV, hepatitis E virus; ORF, open reading frame; qPCR, quantitative PCR; ratHEV, rat hepatitis E virus.

Appendix Table 3. Characteristics of pigs that tested positive for rat hepatitis E virus*

ID no.	Farm code	Aptitude	Breed	Ct value	GenBank accession no.
158	017CO00036	Reproductive	White	33.22	OR977681
159	017CO00036	Reproductive	White	41.32	OR827969
167	017CO00036	Reproductive	White	39.60	OR827970
170	017CO00036	Reproductive	White	39.29	OR977682
171	017CO00036	Reproductive	White	4120	OR827971
175	017CO00036	Reproductive	White	40.91	OR827972
176	017CO00036	Reproductive	White	41.84	OR827973
186	017CO00036	Fattening	White	38.88	OR977683
187	017CO00036	Fattening	White	37.88	OR977684
188	017CO00036	Fattening	White	38.82	OR977685
190	017CO00036	Fattening	White	36.00	OR977686
191	017CO00036	Fattening	White	37.60	OR977687
192	017CO00036	Fattening	White	37.50	OR977688
193	017CO00036	Fattening	White	37.18	OR977689
195	017CO00036	Fattening	White	38.63	OR977690
196	017CO00036	Fattening	White	37.47	OR977691
198	017CO00036	Fattening	White	35.53	OR977692
199	017CO00036	Fattening	White	38.85	OR977693
200	017CO00036	Fattening	White	37.90	OR977694
201	017CO00036	Fattening	White	32.43	OR977695
202	017CO00036	Fattening	White	37.08	OR827964
203	017CO00036	Fattening	White	30.57	OR977696
204	017CO00036	Fattening	White	34.48	OR977697
205	017CO00036	Fattening	White	39.02	OR827965
207	017CO00036	Fattening	White	33.54	OR977698
208	017CO00036	Fattening	White	35.08	OR977699
210	017CO00036	Fattening	White	38.66	OR827976
211	017CO00036	Fattening	White	33.82	OR977700
212	017CO00036	Fattening	White	31.85	OR977701
213	017CO00036	Fattening	White	35.13	OR977702
214	017CO00036	Fattening	White	36.29	OR977703
215	017CO00036	Fattening	White	40.04	OR827977
216	017CO00036	Fattening	White	31.32	OR977704
217	017CO00036	Fattening	White	35.27	OR827978
218	017CO00036	Fattening	White	34.00	OR977705
219	017CO00036	Fattening	White	34.36	OR977706
220	017CO00036	Fattening	White	36.95	OR977707
221	017CO00036	Fattening	White	35.93	OR977708
223	017CO00036	Fattening	White	38.76	OR977709
224	017CO00036	Fattening	White	38.47	OR827979
234	ES140050000005	Reproductive	Iberian Cross	37.06	OR827980
248	ES140050000005	Reproductive	Iberian Cross	37.41	OR827981
265	ES140050000005	Fattening	Iberian Cross	34.70	OR977710
302	ES140050000005	Fattening	Iberian Cross	38.00	OR977711

*Ct, cycle threshold; ID, identification.

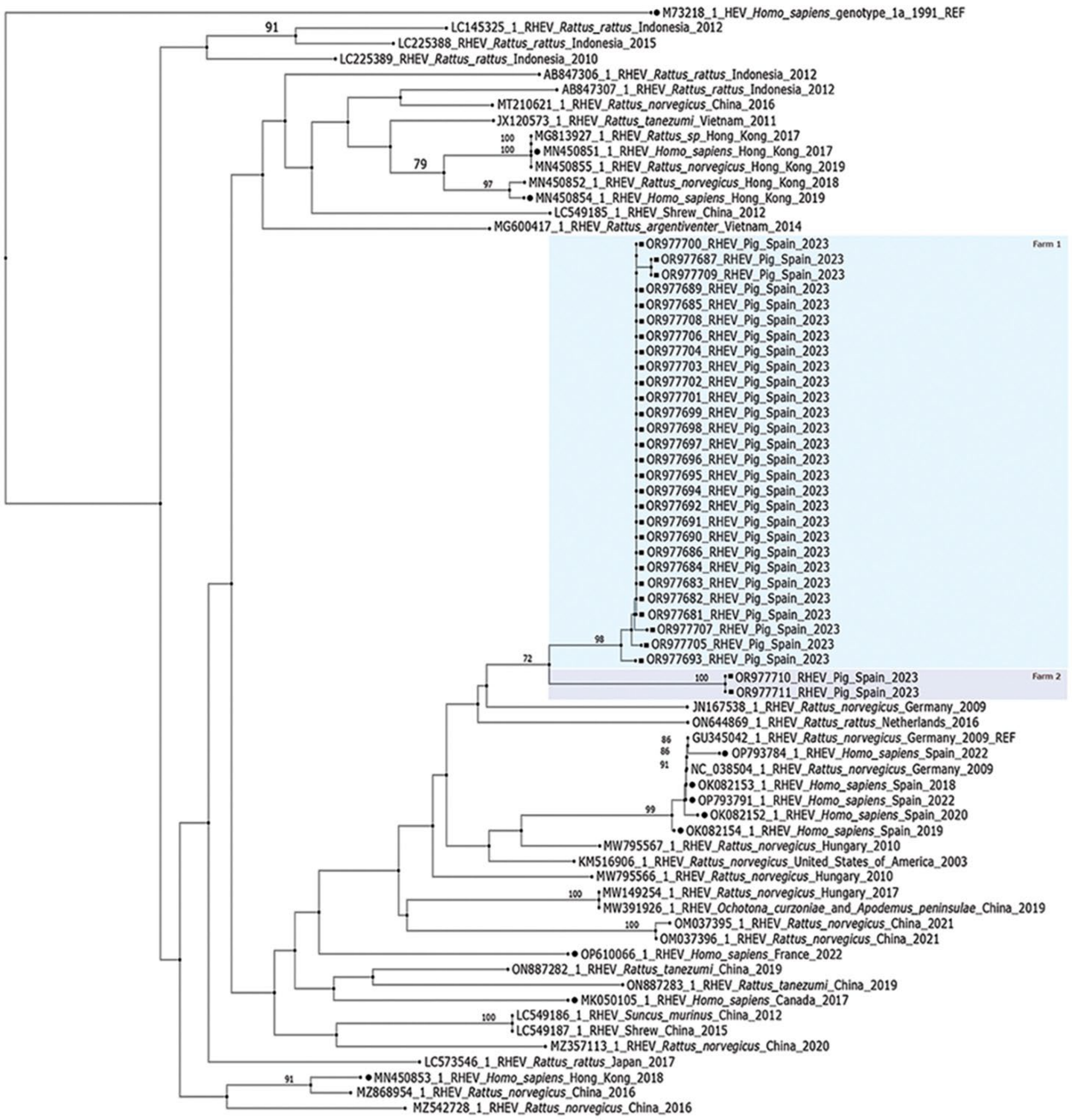

Appendix Figure 1. Phylogenetic analysis of 65 hepatitis E sequences identified in the study. Sequences were 788 nt in length. Squares (■) indicate sequences of pigs identified in this study; circles (●) indicate previously identified human cases. In color is highlight the farm of origin of positive pigs. The evolutionary history was inferred by using the Maximum Likelihood method based on the Tamura-Nei model. The bootstrap consensus tree inferred from 1,000 replicates is taken to represent the evolutionary history of the taxa analyzed.

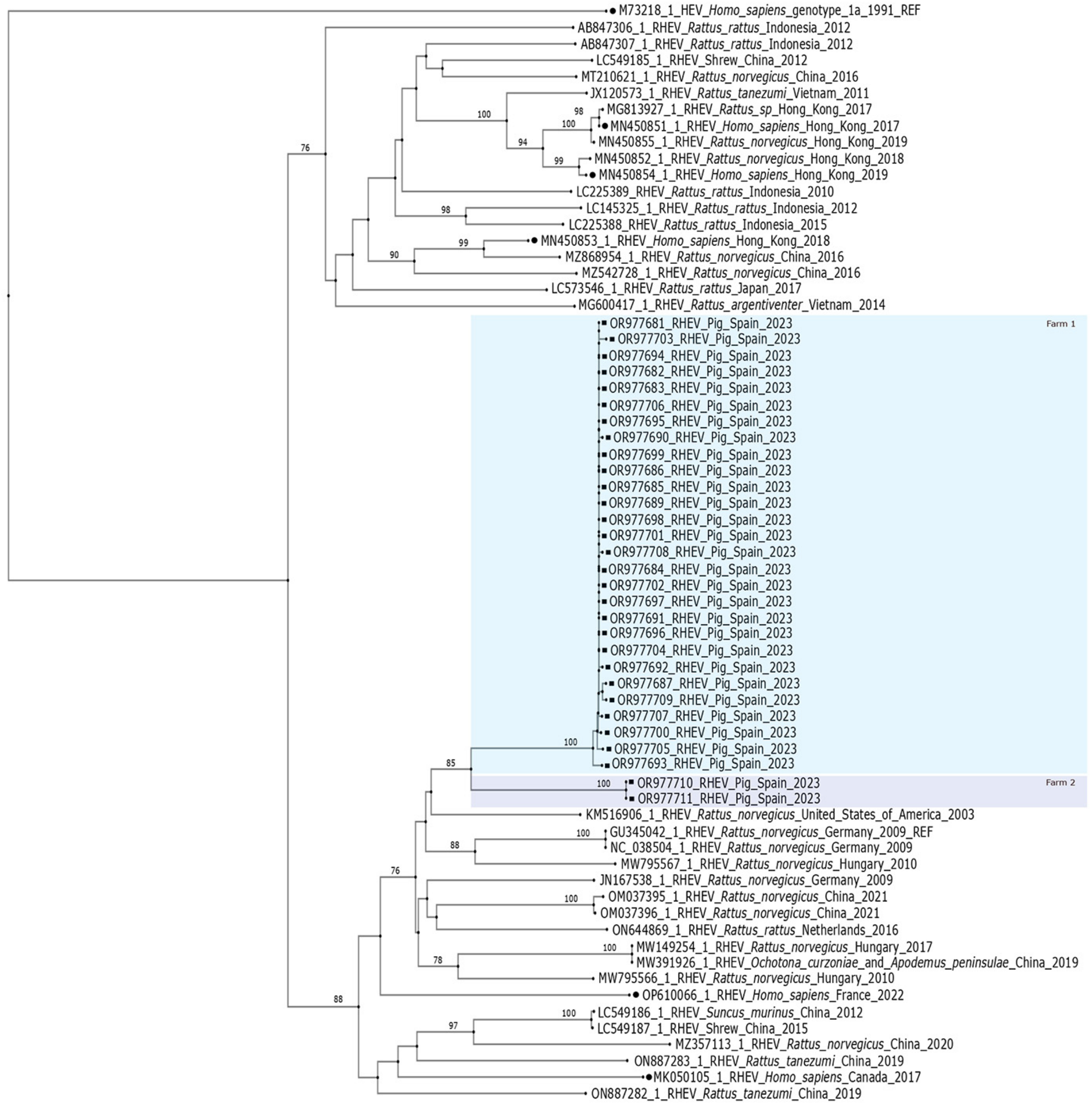

Appendix Figure 2. Phylogenetic analysis of 70 hepatitis E sequences identified in the study. Sequences were 285 nt in length. Squares (■) indicate sequences of pigs identified in this study; circles (●) indicate previously identified human cases. In color is highlight the farm of origin of positive pigs. The evolutionary history was inferred by using the maximum-likelihood method based on the Tamura-Nei model. The bootstrap consensus tree inferred from 1,000 replicates is taken to represent the evolutionary history of the taxa analyzed.
